# An Elusive Diagnosis of Vanishing Bile Duct Syndrome in an HIV Patient

**DOI:** 10.1155/carm/1829205

**Published:** 2025-12-19

**Authors:** Mohammed Ayyad, Radhika Patel, Kevin Molnar, Jared Walsh

**Affiliations:** ^1^ Department of Medicine, Rutgers New Jersey Medical School, Newark, New Jersey, USA, rutgers.edu

**Keywords:** bile duct pathology, HIV, immunosuppression, SIADH, transaminitis, vanishing bile duct syndrome

## Abstract

Vanishing bile duct syndrome (VBDS) is an extremely rare disorder characterized by the progressive destruction of intrahepatic bile ducts, leading to cholestasis and liver dysfunction. It is most commonly associated with autoimmune and infectious conditions but can also occur in immunocompromised states such as human immunodeficiency virus (HIV). We report the case of a 58‐year‐old Hispanic woman with HIV who presented with cyclical fevers, transaminitis, hyperbilirubinemia, and hyponatremia over a 3‐month period. Laboratory workup revealed marked cholestasis with severe elevations in alkaline phosphatase. A liver biopsy confirmed VBDS with a significant reduction in intrahepatic bile ducts. The patient continued antiretroviral treatment, prophylactic antibiotics, and was started on ursodiol, resulting in improvement in liver tests. An extensive workup was done to investigate for an underlying opportunistic infection or lymphoproliferative disease as a culprit for VBDS. VBDS, while exceedingly rare in HIV‐positive patients, is likely immune mediated, involving T‐cell dysfunction and cytokine‐mediated biliary epithelial cell apoptosis. While drug‐induced hepatotoxicity from antiretroviral therapy is a possible contributing factor, it is crucial to consider other underlying etiologies in immunocompromised patients such as infections and lymphoproliferative disorders. VBDS should be considered in HIV‐positive patients with unexplained liver dysfunction, particularly those with low CD4 counts. Early recognition and diagnosis are crucial for initiating appropriate treatment and preventing further liver damage. This case highlights the diagnostic complexity and need for ongoing research into the interplay between immunosuppression, cholangiopathy, and liver dysfunction in HIV patients.

## 1. Introduction

Infection with human immunodeficiency virus (HIV) is increasingly challenging to manage as it progresses to acquired immunodeficiency syndrome (AIDS) because a decreased immune response allows the host to be susceptible to numerous opportunistic infections such as *Pneumocystis jirovecii* pneumonia (PCP), toxoplasmosis, tuberculosis, *Mycobacterium avium* complex, and cryptococcus [1]. The introduction of antiretroviral treatment (ART) significantly decreased the rates of opportunistic infections by as much as 94% for infections such as PCP; however, patients who are not optimized on their ART or are nonadherent remain at an elevated risk of opportunistic infections [2, 3]. Viral load and CD4 cell counts remain the two best prognostic markers to evaluate an individual’s risk of opportunistic infection and can help physicians with managing their patients [4].

Vanishing bile duct syndrome (VBDS) is an exceedingly rare condition characterized by progressive loss of intrahepatic bile ducts, defined as loss of more than 50% of these ducts [5]. While the pathogenesis of VBDS is not well understood, it is thought to be associated with immunological injury likely due to T‐cell reaction causing apoptosis of biliary epithelial cells and is associated with immunocompromised states such as AIDS [5–7]. Although not classic manifestations of HIV/AIDS, hyperbilirubinemia, transaminitis, and hyponatremia all have been reported to be associated with chronic, advanced, or opportunistic infections, as well as medication side effects [8–10]. While VBDS is thought to be rare, the difficult nature of its diagnosis and relatively uncommon presentation may lead to it being underreported [11]. It most often presents with symptoms of jaundice, fatigue, and pruritus. While blood work is usually significant for persistently elevated bilirubin and alkaline phosphatase levels and serum aminotransferases in the normal to near‐normal range, some patients, mainly those with concurrent inflammatory or infectious triggers, may also exhibit transient transaminitis during inflammatory or infectious flares. Confirmation can be attained through biopsy proving at least 50% decrease in intrahepatic bile ducts [7]. The diagnosis can be further complicated by similar presentations of more common diseases such as primary biliary cholangitis, sclerosing cholangitis, and Hodgkin’s lymphoma or the overlapping processes such as hepatic dysfunction or immune dysregulation commonly seen with associated conditions.

In our case, a middle‐aged woman with history of HIV presented with cyclical fevers coinciding with elevations in aminotransferases and direct bilirubin and was subsequently diagnosed with VBDS. Although few cases of VBDS in HIV positive patients are reported, there is no clear understanding of the relationship between the two or how HIV status can impact VBDS progression. We aim to shed light on the diagnostic challenges, possible etiologies, and treatment strategies for managing VBDS in an HIV‐positive patient presenting with cyclical fevers, transaminitis, and hyponatremia.

## 2. Case Presentation

A 58‐year‐old Hispanic female with a history of HIV on bictegravir/emtricitabine/tenofovir alafenamide presented with a 3‐month history of cyclical fever, fatigue, nausea, vomiting, anorexia, and significant weight loss. She reported that these symptoms recurred every 15–20 days, each episode being characterized by high fevers (up to 102°F), worsening epigastric abdominal pain, severe malaise, and oral intolerance to food and liquids. During her most recent exacerbation, she noted progressive weight loss, losing approximately 10 lbs in just five days. She denied recent travel, tuberculosis exposure, or changes to her medication regimen and reported compliance with her antiretroviral therapy. Despite her adherence, her symptoms had progressively worsened.

On physical examination, she appeared fatigued and chronically ill, though not in acute distress. Her vital signs on admission included a blood pressure of 105/80 mmHg, heart rate of 85 beats per minute, respiratory rate of 21 breaths per minute, and a temperature of 97.4°F. Initial laboratory workup revealed significant abnormalities: aspartate aminotransferase (AST) of 75 U/L, alanine aminotransferase (ALT) of 112 U/L, total bilirubin of 1.8 mg/dL with direct bilirubin at 1.3 mg/dL, and a markedly elevated alkaline phosphatase (ALP) of 1465 U/L. Her most recent CD4 count was 31 cells/mm^3^, her HIV viral load was undetectable, and serum sodium was low at 125 mEq/L. Review of prior laboratory data revealed persistently low CD4 counts, ranging from 28 to 45 cells/mm^3^ over the preceding year despite viral suppression. These levels remained stable throughout her febrile episodes, with no clear correlation between CD4 trajectory and LFT fluctuations, making immune reconstitution inflammatory syndrome unlikely.

Given her low CD4 count and recurrent fevers, she was empirically started on broad‐spectrum antibiotics, including vancomycin, cefepime, and atovaquone. Comprehensive infectious, hematological, and oncological workups were initiated. A chest computed tomography (CT) scan revealed diffuse bilateral pulmonary nodules, mediastinal and retroperitoneal lymphadenopathy, and splenomegaly, raising concern for infection or malignancy (Figure 1). Acid‐fast bacillus cultures from her sputum were positive in 1 of 3 samples; however, GeneXpert testing for *Mycobacterium tuberculosis* was negative, serial mycobacterial cultures failed to yield growth, and bronchoscopic evaluation including transbronchial biopsies was nondiagnostic, making active mycobacterial infection less likely. Additional infectious testing for cytomegalovirus (CMV) and fungal antigens was unremarkable. However, her EBV polymerase chain reaction (PCR) was significantly elevated at 90,490 IU/mL, raising suspicion for EBV‐associated lymphoproliferative disease.

Figure 1Chest CT scan showing diffusely enlarged mediastinal lymph nodes, including (a) a right paratracheal node measuring 1.3 cm, (b) a subcarinal node measuring 1.5 cm, and (c) a right hilar node measuring 1.7 cm.

**Figure:**
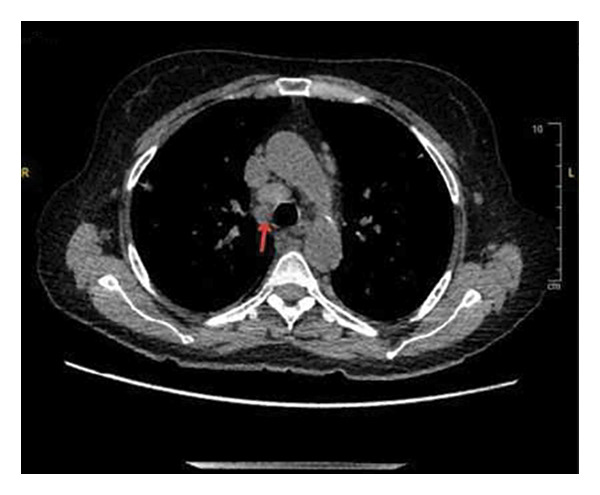
(a)

**Figure:**
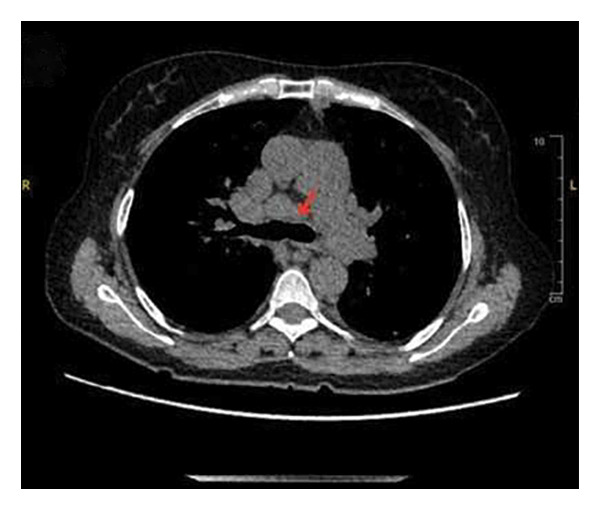
(b)

**Figure:**
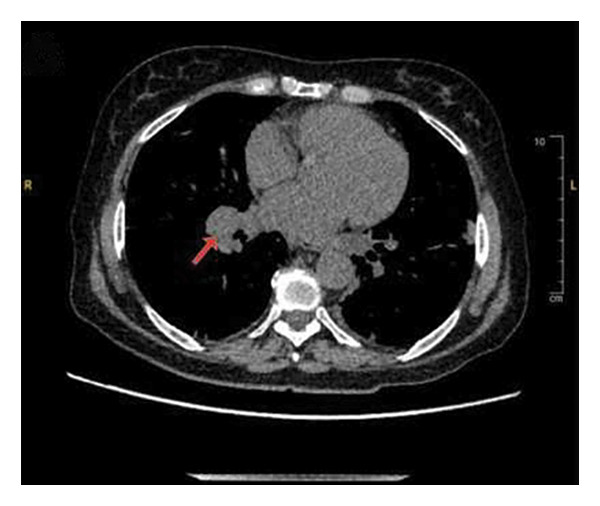
(c)

During her hospital stay, it became evident that her liver function abnormalities and hyponatremia followed a cyclical pattern, worsening in parallel with her fever episodes. When her fevers spiked, her liver enzymes, bilirubin, and ALP would rise sharply. AST peaked at 192 U/L, ALT reached 159 U/L, total bilirubin rose to 3.5 mg/dL, and ALP spiked at 1899 U/L—significantly worse than her admission values. Additionally, a similar pattern of hyponatremia was noted during her hospitalization. During each fever episode, her serum sodium would drop as low as 125 mEq/L and would spontaneously normalize to 141 mEq/L after the fevers resolved. Initially, the hyponatremia was thought to be due to dehydration, and intravenous fluids were administered. However, this led to a further drop in serum sodium to 123 mEq/L, prompting further investigation with serum and urine osmolality studies. Subsequently, her serum osmolality was found to be low at 247 mOsm/kg, while urine osmolality was elevated at 871 mOsm/kg, confirming the diagnosis of syndrome of inappropriate antidiuretic hormone secretion (SIADH). The patient was subsequently managed with fluid restriction, leading to gradual improvement in sodium levels. Serial inflammatory markers were also followed during the hospitalization. Although daily values were not consistently obtained, ESR was repeatedly elevated during febrile episodes (peaking > 145 mm/hr) and decreased during afebrile intervals (range: 73–101 mm/hr).

**Figure 3:**
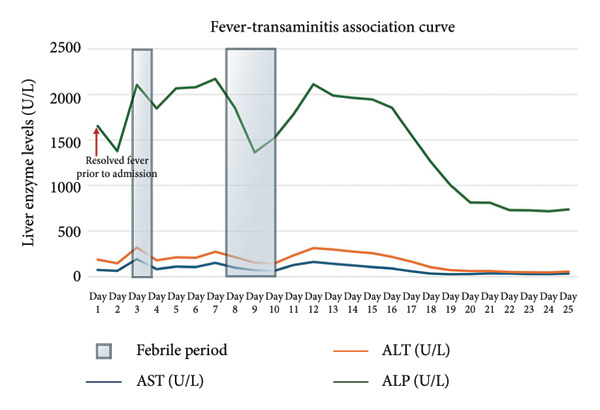
Fever–transaminitis association curve. Longitudinal trends of liver function tests during hospitalization. Alkaline phosphatase (ALP, green), alanine aminotransferase (ALT, orange), and aspartate aminotransferase (AST, blue) are plotted across 25 days. Shaded intervals denote febrile periods. The figure illustrates a predominantly cholestatic pattern with persistently elevated ALP, punctuated by transient spikes in AST and ALT during febrile episodes. Enzyme levels normalized after fever resolution, whereas ALP remained disproportionately elevated, consistent with vanishing bile duct syndrome (VBDS).

**Figure 4:**
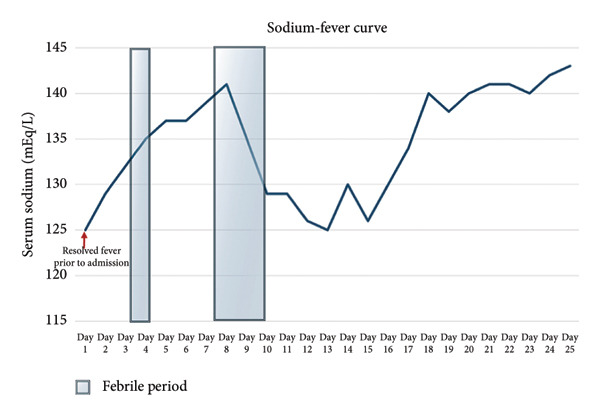
Fever‐sodium trends during hospitalization. Longitudinal serum sodium values (mEq/L) over 25 days. Shaded regions represent febrile periods. Sodium dropped acutely during febrile episodes and normalized during afebrile intervals, consistent with cyclical syndrome of inappropriate antidiuretic hormone secretion (SIADH). The red arrow denotes the resolution of a fever episode just prior to hospital admission.

Given the cholestatic liver function abnormalities and hyperbilirubinemia, an MRI of the abdomen was performed, revealing no obstructive cause of cholestasis, though there was notable periportal and hilar lymphadenopathy, raising suspicion for a more insidious intrahepatic process (Figure 2). Subsequently, an endoscopic retrograde cholangiopancreatography (ERCP) was performed, which demonstrated absence of mechanical obstruction, nonvisualization of several intrahepatic ducts, and irregular tapering of peripheral ducts, findings supportive of ductopenia. A subsequent liver biopsy confirmed the presence of VBDS, with histopathology demonstrating ductopenia and bile duct paucity in the portal tracts, along with a mixed inflammatory infiltrate composed primarily of lymphocytes and eosinophils. Notably, no significant interface hepatitis or steatosis was observed. The biopsy findings confirmed the suspicion of VBDS. In response to the liver dysfunction, the patient was continued on her antiretroviral therapy and ursodiol 250 mg three times daily was initiated. Over the course of several days, her liver function tests (LFTs) began to improve. However, her improvement was interrupted by another episode of cyclical fevers and constitutional symptoms, where it was noted that her LFTs began to rise again and hyponatremia worsened, as shown in Figures 3 and 4. The patient was continued on ursodiol, and the episode was treated conservatively with antipyretics and fluid restriction for suspected SIADH. After the fever episode resolved, her ALP trended down again from its peak of 1899 to 681 U/L by the time of discharge, and her total bilirubin normalized from 3.5 to 0.8 mg/dL. The cyclical transaminitis associated with her fever episodes also resolved, with AST and ALT normalizing in parallel with the resolution of her fevers.

Figure 2MRI of the abdomen without contrast. (a) Axial MRI image demonstrating right hilar lymphadenopathy measuring approximately 3.1 cm. (b) Axial MRI showing retroperitoneal and mesenteric lymphadenopathy, with multiple nodes measuring up to 1.0 cm.

**Figure:**
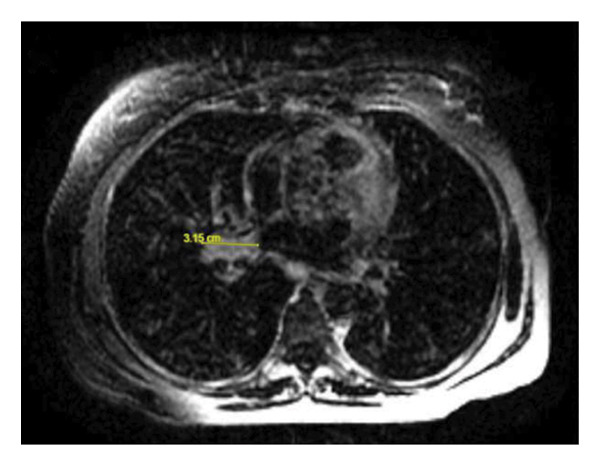
(a)

**Figure:**
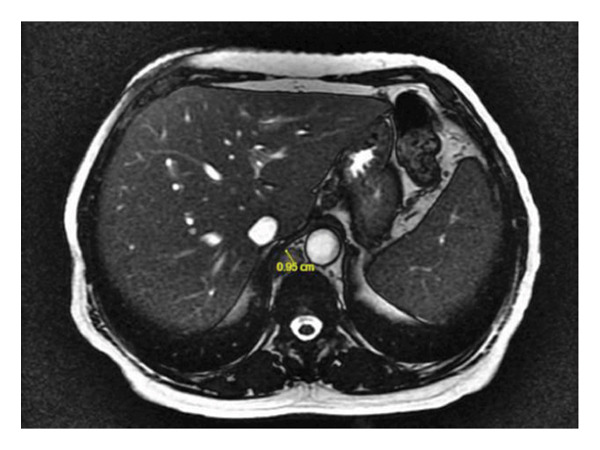
(b)

Despite the extensive workup, no definitive cause of her symptoms was identified. Subsequently, the patient underwent bronchoscopy with endobronchial ultrasound and transbronchial biopsies of mediastinal and hilar lymph nodes to evaluate for malignancy or granulomatous infection, which revealed rare atypical cells but were otherwise negative for malignancy or granulomatous disease. A left lower lobe lung biopsy revealed alveolated lung parenchyma with focal lymphohistiocytic inflammation and features suggestive of lipoid pneumonia. No granulomas were identified. Special stains for mycobacterial (AFB) and fungal (GMS) organisms were negative, and EBV in situ hybridization was also negative.

After her laboratory values normalized and her fever episodes abated, the patient was deemed stable for discharge. She was discharged on ursodiol and advised to continue fluid restriction to manage SIADH. At follow‐up visits, the patient reported improvement in her symptoms, including a decrease in the frequency and severity of her cyclical fevers and abdominal pain. However, the long‐term prognosis remained uncertain due to the potential progression of her VBDS and the possibility of an underlying lymphoproliferative disorder, given her history of low CD4 count and elevated EBV PCR levels. Further investigations, including repeat imaging and possible lymph node biopsy, were planned as part of her ongoing care.

## 3. Discussion

The case presented a 58‐year‐old Hispanic woman with HIV, managed on bictegravir/emtricitabine/tenofovir alafenamide with an undetectable viral load and a CD4 count of 31, who experienced cyclical fevers accompanied by transaminitis, hyperbilirubinemia, and hyponatremia. Upon admission, the patient’s labs were significant for transaminitis and an alkaline phosphatase level of 1465 U/L. The hepatic biopsies confirmed VBDS, with histology demonstrating marked ductopenia and mild portal inflammation. The absence of granulomas, viral inclusions, or significant interface hepatitis supported an immune‐mediated or idiopathic process rather than direct infectious infiltration. Although VBDS typically presents with cholestatic liver enzyme abnormalities, our patient demonstrated intermittent elevations in aminotransferases coinciding with febrile episodes and systemic inflammatory flares. This mixed pattern has been reported in the setting of concomitant infections, neoplasms, or inflammatory triggers in immunocompromised patients and does not preclude the diagnosis of VBDS, as the underlying process remained cholestasis predominant. The patient’s symptoms, characterized by recurrent high fevers every 15–20 days, coincided with hyponatremia, leading to the clinical diagnosis of cyclical SIADH.

VBDS, though exceedingly rare, poses unique challenges in the context of HIV infection. One proposed mechanism is immune‐mediated destruction of bile ducts, driven by CD8+ cytotoxic T‐cell activation and cell death–mediated cytokine release, which could occur in AIDS due to HIV‐associated immune dysregulation inducing biliary epithelial cell apoptosis [5]. Drug‐induced hepatotoxicity, on the other hand, while more commonly linked to antibiotics and psychiatric medications, has also been reported in HIV patients on antiretroviral therapy, with one documented case of nevirapine‐associated VBDS. Although this patient’s antiretroviral regimen did not include nevirapine, the potential for liver injury from medications remains a reasonable consideration [12, 13].

Current literature suggests that patients with advanced HIV are at increased risk for liver‐related complications, including various forms of cholestasis due to drug‐mediated liver injury, opportunistic infections, or the use of antimicrobial agents [14]. Previous case reports have noted VBDS in HIV‐positive individuals, indicating a potential association, though the underlying mechanisms remain poorly understood [5]. This case highlights the need for vigilance in monitoring liver function in patients with low CD4 counts, as this population may experience atypical liver pathologies, some of which can be acutely life threatening.

On the other hand, the patient’s cyclical fever pattern presents intriguing questions regarding the underlying mechanisms at play. Such episodes may reflect immune system activation triggered by opportunistic infections or inflammatory responses to viral reactivation, notably EBV, which was markedly elevated in this case [15]. Similar instances in the literature highlight the correlation between cyclical fever, transaminitis, and cholestasis, often observed in patients with Hodgkin’s lymphoma [16]. Further exploration into these episodic exacerbations may reveal critical insights into the pathophysiology of liver dysfunction in this population. Supporting this, inflammatory markers such as ESR demonstrated cyclical elevations in close temporal relation to fever and liver enzyme flares, with peaks exceeding 145 mm/hr and subsequent downtrends during afebrile periods. This dynamic inflammatory pattern, although not captured with daily CRP or ferritin values, reinforces the interpretation that the patient’s VBDS course was immune‐mediated rather than solely drug‐ or infection‐driven.

Although one of three sputum samples was AFB smear positive, GeneXpert testing was negative for *Mycobacterium tuberculosis*, serial mycobacterial cultures showed no growth, and both bronchoscopy with transbronchial biopsies and a subsequent lung biopsy were nondiagnostic for mycobacterial infection. Furthermore, AFB and GMS stains on lung tissue were negative. Taken together, these findings argue strongly against active mycobacterial disease and reduced the rationale for empiric antitubercular therapy. While initial suspicion leaned toward opportunistic infections such as CMV, negative titers shifted the differential toward a possible underlying lymphoproliferative disorder. Despite performing an inguinal lymph node biopsy, a definitive diagnosis of lymphoma could not be made due to difficulties obtaining an appropriate tissue sample. Subsequently, a lung tissue biopsy obtained via endobronchial ultrasound was performed and showed evidence of lipoid pneumonia, although no concrete evidence of infection or lymphoproliferative disease. Importantly, the decision to pursue an ERCP and liver biopsy was pivotal in confirming VBDS and in guiding subsequent investigation for its underlying cause in our immunocompromised patient.

The management of this patient included the continuation of antiretroviral therapy and the initiation of ursodiol, which effectively addressed her cholestatic liver function abnormalities. A conservative approach revolving around fluid restriction was used to address the patient’s cyclical SIADH. The reduction in liver enzymes and bilirubin levels upon treatment and the resolution of fever episodes and hyponatremia indicate a positive response though the cyclical nature of her symptoms raises concerns about long‐term management. VBDS can lead to progressive liver damage, particularly in the context of HIV‐related immunosuppression, which necessitates close monitoring and potential interventions to address any evolving complications [17].

This case highlights the need for clinicians to remain alert to the possibility of VBDS in HIV patients, particularly those with low CD4 counts and unexplained liver dysfunction. Enhanced awareness may facilitate earlier diagnosis and treatment, potentially improving outcomes. Furthermore, once VBDS is confirmed, it is crucial to investigate for an underlying inflammatory, infectious, or neoplastic cause, as they are common triggers for VBDS in this patient population. Future research should focus on elucidating the relationship between immunosuppression, bile duct pathology, and liver dysfunction in this demographic, as well as developing specific guidelines for managing complex cases that present with cyclical liver dysfunction.

## Disclosure

All authors read and approved the final manuscript.

## Conflicts of Interest

The authors declare no conflicts of interest.

## Author Contributions

Mohammed Ayyad contributed to the conceptualization, investigation, data curation, writing of the original draft, and critical review and editing of the manuscript. Radhika Patel contributed to the investigation, literature review, writing of the original draft, and editing of the manuscript. Kevin Molnar contributed to the literature review, writing of the original draft, and editing of the manuscript. Jared Walsh supervised the study, provided critical review, and approved the final version of the manuscript.

## Funding

No funding was received for this research.

## Data Availability

The data used to write this case report are available from the corresponding author upon reasonable request.
